# Large-Scale Transcriptome Analysis in Faba Bean (*Vicia faba* L.) under *Ascochyta fabae* Infection

**DOI:** 10.1371/journal.pone.0135143

**Published:** 2015-08-12

**Authors:** Sara Ocaña, Pedro Seoane, Rocio Bautista, Carmen Palomino, Gonzalo M. Claros, Ana M. Torres, Eva Madrid

**Affiliations:** 1 Área de Mejora y Biotecnología, IFAPA Centro Alameda del Obispo, Apdo 3092, E-14080, Córdoba, Spain; 2 Departamento de Biología Molecular y Bioquímica, Universidad de Málaga, E-29071, Málaga, Spain; 3 Plataforma Andaluza de Bioinformática, Universidad de Málaga, E-29071, Málaga, Spain; 4 Institute for Sustainable Agriculture, CSIC, Apdo 4084, E-14080, Córdoba, Spain; National Key Laboratory of Crop Genetic Improvement, CHINA

## Abstract

Faba bean is an important food crop worldwide. However, progress in faba bean genomics lags far behind that of model systems due to limited availability of genetic and genomic information. Using the Illumina platform the faba bean transcriptome from leaves of two lines (29H and Vf136) subjected to *Ascochyta fabae* infection have been characterized. *De novo* transcriptome assembly provided a total of 39,185 different transcripts that were functionally annotated, and among these, 13,266 were assigned to gene ontology against *Arabidopsis*. Quality of the assembly was validated by RT-qPCR amplification of selected transcripts differentially expressed. Comparison of faba bean transcripts with those of better-characterized plant genomes such as *Arabidopsis thaliana*, *Medicago truncatula* and *Cicer arietinum* revealed a sequence similarity of 68.3%, 72.8% and 81.27%, respectively. Moreover, 39,060 single nucleotide polymorphism (SNP) and 3,669 InDels were identified for genotyping applications. Mapping of the sequence reads generated onto the assembled transcripts showed that 393 and 457 transcripts were overexpressed in the resistant (29H) and susceptible genotype (Vf136), respectively. Transcripts involved in plant-pathogen interactions such as leucine rich proteins (LRR) or plant growth regulators involved in plant adaptation to abiotic and biotic stresses were found to be differently expressed in the resistant line. The results reported here represent the most comprehensive transcript database developed so far in faba bean, providing valuable information that could be used to gain insight into the pathways involved in the resistance mechanism against *A*. *fabae* and to identify potential resistance genes to be further used in marker assisted selection.

## Introduction

Faba bean (*Vicia faba* L.), one of the first domesticated plant species in Old World agriculture, is an important food crop worldwide and a source of dietary protein in developing countries. It ranks fourth in terms of cultivation after key food legumes such as chickpea, pea and lentil (http://faostat.fao.org). World production of dry faba beans remained stable in the last five years (4.2 million tons), with China and Ethiopia producing almost half. Traditionally grown in the Mediterranean basin, Middle East, China and Latin America the crop is currently achieving particular relevance in Australia (third world producer), Europe and North America. The main faba bean breeding objectives today include yield and seed quality improvement as well as resistance to biotic and abiotic stresses. Among them, broomrape (*Orobanche crenata*), a highly damaging parasitic weed, and Ascochyta blight, caused by *Ascochyta fabae* Speg., are the most serious threats to faba bean cultivation in most parts of the world.

The wild faba bean progenitor is unknown, and crosses with other *Vicia* species have proved unsuccessful [1, 2], thus limiting genetic diversity to the primary gene pool. Faba bean has a diploid set of 12 chromosomes and a genome of 13,000 Mbp [3] with a high proportion of repetitive DNA elements [4]. It represents, one of the largest legume genomes, exceeding 25 times that of the model legume *Medicago truncatula*. These biological aspects are the major challenges towards faba bean genome sequencing and associated marker development for efficient genomics-assisted breeding. In spite of these limitations, saturated genetic maps built with RAPDs (Random Amplified Polymorphic DNA), SSRs (Simple Sequence Repeats) and gene-based SNPs (Single Nucleotide Polymorphism) markers from *M*. *truncatul*a, pea (*Pisum sativu*m L.), lupin *(Lupinus albu*s L.), lens *(Lens culinaris* Medik.) and soybean (*Glycine max* L. Merr), are now available [5–9]. These maps allowed the location of genes and QTLs (Quantitative Trait Loci) controlling faba bean resistance to different pathogens such as broomrape [10–12], *A*. *fabae* [13–17], rust [14] and other important agronomic traits as yield or drought adaptation [9, 18]. Nevertheless, the genomic regions controlling those characters are still not well-characterized and no diagnostic markers have been developed either. So, the identification of markers with complete association with the QTL will boost the development of “perfect” markers in pulses [19]. Such markers are extremely useful for guiding the introgression of multiple resistant genes, because they increase selection efficiency and avoid recombination events between markers and QTLs [20].

The success of marker assisted selection (MAS) for agronomically important traits will depend on the extent to which the underlying genes and pathways are identified and further used for the development of candidate gene markers [21]. However, faba bean genomics lags far behind that of model systems. This is reflected by the low number of faba bean ESTs (Expressed Sequence Tags) present in the dbEST database at NCBI (release 130101; 1 January 2013). Only 5,510 ESTs are available for this crop, compared with 1.5 million and 270,000 public entries for *Arabidopsis* and *M*. *truncatula* ESTs, respectively (http://www.ncbi.nlm.nih.gov/dbEST). Therefore, much effort is still needed to develop comprehensive faba bean genomic resources and to generate fast and cost-effective gene-based markers useful for molecular breeding or comparative genomic analysis.

The emergence of next generation sequencing (NGS) technology in the latest decade, is rapidly changing this scenario [22]. Compared to traditional sequencing methods, NGS yields a large number of sequences suitable to develop fast and cost-effective gene-based markers such as EST-based SSRs, SNPs or Indels (short insertions and deletions) and therefore, high-density genetic maps. Examples in faba bean include 2,397 [23] and 28,503 SSRs [24] that are already available. Moreover, SNP assays using the KASPar (KBiosciences Competitive Allele Specific PCR) platform (LGC, UK) have been described [25], and have been used to build a fairly dense genetic map for genomic-assisted breeding [9]. Besides marker development, NGS offers an efficient way for wide transcriptome assemblies in crops with large and poorly characterized genomes, as faba bean is [26]. This approach provides an excellent resource of gene sequence information and expression data for identification of candidate genes associated with relevant traits. A preliminary faba bean transcriptome analysis [23] identified a collection of EST-derived SNPs that were used to develop a genetic map in a faba bean population segregating for ascochyta blight resistance [16].

In order to increase the publicly available genomic data for this crop and to further assist in the development of allele-specific markers efficient in breeding selection, we performed a comprehensive characterization of the faba bean transcriptome derived from leaves of two lines (29H and Vf136) subjected to *A*. *fabae* infection, using the Illumina platform. Illumina reads together with the 105,094 faba bean sequences available in public databases were used for *de novo* transcriptome assembly. Results reported here will aid in identifying potential gene targets for use in marker assisted selection and represent the most comprehensive transcripts database reported so far in this crop species.

## Material and Methods

### Plant material and *A*. *fabae* inoculation

Two faba bean genotypes (29H and Vf136) were used for de novo transcriptome assembly. Line 29H has been described as resistant to *A*. *fabae* in several studies [27–30] and Vf136 belongs to the germplasm collection of the IFAPA Centro Alameda del Obispo in Córdoba (Spain), and is susceptible to ascochyta blight [17].

Seeds of both genotypes were pre-germinated and sown with three replicates; three plants each, in 14 cm-diameter pots, using a 1:1 mixture of sand and peat. All plants were grown in a controlled condition chamber at 20–22°C. Inoculation was performed as described by Madrid et al. [31] using a monoconidial isolate of *A*. *fabae*, CO99-01, originating from Córdoba, Spain. Non-inoculated replicated plants were included in the assay as controls. To confirm that *A*. *fabae* infection had been effective, inoculated plants were checked for expected disease symptoms at 15 days after inoculation and compared to controls.

### RNA extraction, library preparation and DNA sequencing

As early defence responses occur shortly after contact with a pathogenic organism [32, 33], entire leaf tissue was taken from plants at 4, 8 and 12 hours after inoculation, and immediately frozen in liquid nitrogen. An identical collection of leaf tissue was accomplished in the non-inoculated plants of the same lines. Total RNA isolation and cDNA synthesis were performed as described in a previous study [31]. Two cDNA libraries were generated from the pooled time point RNAs of each faba bean line (29H and Vf136). The library construction and sequencing was performed by GenXPro, Frankfurt am Main, Germany. Normalization of cDNA was performed as described by Zhulidov et al. [34] and Bogdanova et al [35]. The normalized cDNA was random fragmented and adapters for Illumina sequencing ligated. Products were amplified and size selected on agarose gel. Insert size of the fragments was 150–300 bp. Sequencing was performed on an Illumina GAII machine, using 1 x 100 bp reads. The sequence data generated in this study have been deposited at SRA (Short Read Archive) database under Experiment Accessions SRX690543 (29H) and SRX690544 (Vf136).

### Sequence pre-processing, *de novo* assembly and annotation

With the aim of constructing a faba bean transcriptome that integrates all known sequences in this species, 19,067 entries from European Nucleotide Archive (ENA), and 79,657 singletons and 6,370 contigs from Kaur et al. [23] were incorporated. Illumina reads and these additional faba bean sequences were assembled following an updated version of the workflow described in Seoane et al. [36]. Differences with respect to the published pipeline are that (i) more ENA entries were incorporated, (ii) the pre-processing included more putative contaminant sources of microorganisms, (iii) Full-LengtherNext analyses can now detect and split chimeric transcripts, (iv) UniProtKB database for Full-LengtherNext analyses was updated on September 4^th^, 2014, and (v) the prediction of species-specific coding transcripts was carried out with TransDecoder (http://transdecoder.github.io). The resulting assembling was named as the v 1.1 of the *V*. *faba* transcriptome.

In addition to the annotations provided by Full-LengtherNext in the pipeline, the reconstructed transcripts were compared against the nucleotide sequence database TAIR 10 of *Arabidopsis* orthologue using BLASTN [37] with a threshold *E*-value of 10^−10^. These orthologue identifiers were used for functional analyses using the AgriGo tool (http://bioinfo.cau.edu.cn/agriGO) [38]. A nucleotide comparison using BLASTN was also performed against genetically related legume species (*M*. *truncatula*, and *Cicer aritenum*). Finally, the protein sequences derived from the faba bean transcriptome were compared against the nucleotide sequence database of chickpea using the tBLASTX (*E*-value10^-10^) to derive other putative annotations. The resulting annotated transcriptome is provided in S1 File.

### Variant analyses

Identification of nucleotide variations (SNPs and InDels) was performed by separated mapping data from each genotype against the tentative transcripts using Bowtie v2.1.0 [39]. SNPs were called using bcftools and filtered using vcfutils from the [40], using the default parameters, to obtain a subset of high quality SNPs and InDels.

### Differential gene expression and functional enrichment

The sequence reads obtained from resistant and susceptible genotypes were mapped separately against the reference transcriptome and then counted using Sam2counts.py. The count chart was loaded into the RobiNa software [41] and analyses with the statistical method edgeR [42]. Reliability of differential expression required a *P*-value < 0.05. Finally, a functional enrichment analysis was conducted using Kobas 2.0 (http://kobas.cbi.pku.edu.cn/home.do) with the Fisher's exact test based on the differentially expressed genes detected and known KEGG pathways, in order to identify genes potentially associated with phenotypic differences between lines.

### Expression profiles via RT-qPCR

Two μg of total RNA was reverse transcribed in duplicates from separate tissue samples for each time point from both genotypes and inoculated/non-inoculated plants using the M-MLV reverse transcription enzyme (Invitrogen, Carlsbad, CA, USA), in combination with oligodT (dT12–18) according to manufacture’s instructions. cDNA syntehesis and quality controls were performed as described previously by Madrid et al. 2013. Primers were designed based on their differential expression between both genotypes using the software package Primer 3 (http://frodo.wi.mit.edu/primer3/; Table 1) [43].

**Table 1:** Primer pairs designed to validate RNAseq data by RT-qPCR and number of reads detected in each of the faba bean genotypes.

Gene homology	Primer ID	Forward primer (5'-3')	Reverse primer (5'-3')	Vf 136 Reads	29H Reads
Transcription factor NAI1	C-3026	GGAATCCGGAGAAAATTGGCC	ATTCGAGCCAGGAATGGTGG	2175	0
LEA-18 protein	C-15319	GGAACCATTGAAGGGCTTGC	GAGTAGGTGCCTCAGTTGCA	110	0
Jasmonate O-methyltransferase	L-45566	GTGCAACACCAGGCAGTTTT	TGAGCAAATTTTCTGGCGCC	1	121
F-box/LRR-repeat protein At3g59250	L-57706	CGGTTCACCACTTGGAGTGT	ATGCATTGCCGAAACCACAC	1	291

In order to normalize the data two reference genes, actin1 (ACT1) and cyclophilin (CYP2), were used [44]. The real-time quantitative PCR (qPCR) reactions used the iTaq Universal SYBR (Biorad) according to manufacturer's instructions. qPCR amplifications were carried out in a 7500 HT sequence detection System (Applied Biosystems, Foster City, CA, USA) with the following temperature profile: initial denaturation at 95°C for 5 min, followed by 30 cycles of 95°C for 30 s and 60°C for 30 s (annealing and elongation). No-template controls were included. Amplicon quality was checked by an additional melting curve gradient with fluorescence measures after each temperature step. The amplification of the target genes at each cycle was monitored by SYBR green fluorescence. The Ct, defined as the PCR cycle at which a statistically significant increase of reporter fluorescence is first detected, was used as a measure for the starting copy numbers of the target gene. The efficiency of each primer pair was checked for all templates using LinReg software. The qPCR data were normalized with the relative efficiency of each primer pair.

## Results

### A new faba bean transcriptome

The construction of a complete faba bean transcriptome is the first step towards the identification of putative genes associated with resistance to *A*. *fabae* in this crop. Using RNA obtained from leaf tissue taken at 4, 8 and 12 hours after inoculation, a total of 33,023,160 raw sequences, with a read length of approximately 100 pb were generated. Approximately half of the sequences (16,567,244) belong to the 29H parental line and the other half (16,455,916) to line Vf136. A total of 30,907,202 (93.59%) useful reads were submitted to different assembly procedures as described in Seoane et al. [36]. Additionally, 105,094 sequences from ENA and Kaur et al. [23] were pre-processed to provide 87,269 useful reads to be assembled and merged with the previous assemblies. As a result, 98,195 tentative transcripts were obtained, with the longest contig having 5,439 nucleotides in length (Table 2). This figure is overestimating the number of genes in *V*. *faba*, although the number has been reduced from the previous version (v 1.0), indicating that the new ENA sequences have served to assemble previously fragmented contigs. The percentage of transcripts with annotation (40.12% in v 1.1; S1 File) did not differ significantly between the three transcriptomes, but the number of non-redundant orthologues increased, possibly as a result of the higher input of reads and the improvement of Full-LengtherNext. The 21,243 non-redundant orthologues in Table 2 are closer to the number of total genes in plants, as suggested for *Arabidpsis* (~27,200) [45].

**Table 2:** Summary of the attributes assemblies obtained using three different datasets after the analysis using Full-LengtherNext. Transcriptomes of Kaur et al. [23] and version 1.0 were analysed with older releases of Full-LengtherNext, while the current transcriptome (version 1.1) was analysed with a new release of this software.

Transcriptome attributes	Kaur et al. [23]	v 1.0 [36]	v 1.1 (this work)
Tentative transcripts	85,844	118,188	98,195
Transcripts with annotation	41,049	38,004	39,185
Unique IDs	18,871	20,413	21,243
Transcripts including a complete ORF	1,578	10,516	9,325
Unique complete ORFs	1,424	6,787	6,593
ncRNAs	134	2,789	2,254
Transcripts without orthologue	44,661	77,395	59,010
Coding	0	3,314	270
Putative coding	0	-	579
BA index	0,95	0,86	0,91

From the 39,185 annotated transcripts, 44% (9,325) contained a complete open reading frame for a known protein, 6,593 being non-redundant full-length proteins. The numbers recorded are much higher than those provided by Kaur et al. [23], but do not differ from v 1.0. A detailed analysis indicated that v 1.0 was contaminated with transcripts from microorganisms that are absent in v 1.1 (results not shown).

Although RNA-seq libraries were not designed to detect any ncRNA, due to the deep analysis provided by the Illumina Platform 2,254 putative ncRNAs (including microRNA precursors) were detected by comparing against miRBase [46], although these were not confirmed. Interestingly, a previous transcriptome report identified 134 putative ncRNAs by the same comparison criteria [23].

The proportion of unknown transcripts was 60% in v 1.1, compared to 65% in v 1.0 and 52% in Kaur et al. [23]. This indicates that the update of sequences and software in the workflow described in Seoane et al. [36] clearly improved the faba bean transcriptome. These transcripts can be faba/legume-specific transcripts (without a clear orthologue in the databases) or assembling artefacts. The TransDecoder analyisis included in Full-LengtherNext revaled that 270 transcripts likely are faba/legume-specific and coding for a complete, unknown protein, while 579 transcripts could be coding for a complete protein or a protein fragment.

Based on the characteristics of the transcriptome (Table 1), the non-redundant *V*. *faba* protein-encoding transcriptome consists of 22,092 transcripts (21,243 unique orthologues, 6,593 of them coding for a complete, known protein and 270 + 579 for unknown transcripts). The subset of 21,243 non-redundant known transcripts was selected for further studies. This subset corresponded to the longest transcripts with different orthologous IDs, including those that code for a complete open reading frame (ORF).

### Functional classification of the *V*. *faba* transcriptome

**Fig 1:**
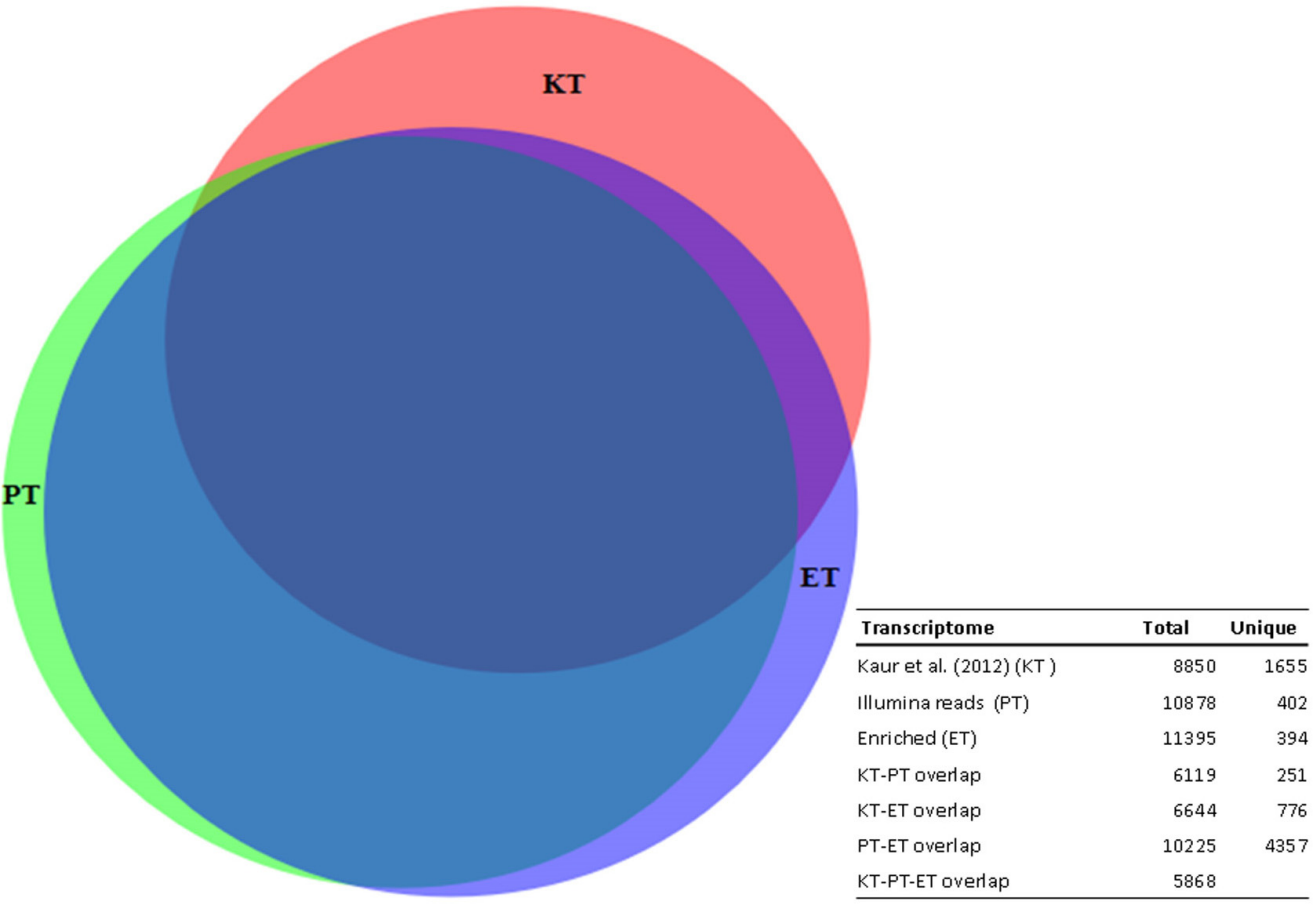
Venn diagram depicting the number of overlapping sequences and unique genes present among the three transcriptomes. KT: transcriptome developed by Kaur et al. [23]; PT: Illumina reads from the present study; ET: enriched transcriptome (Illumina reads and database sequences).

A comparison among previous transcriptome data (KT) [23] with the present transcripts assembled only from Illumina reads (PT) or enriched with database sequences (ET) was performed. These data were obtained from intermediary steps of the assembly workflow (results not shown). The *Arabidopsis* orthologues in the three subsets (KT, PT and ET) were used to assess the overlapping information with BioVenn [47]. This comparison, revealed that the ET transcriptome represents 75% of KT [23] and 95.5% of PT. The Venn diagram depicts the number of overlapping sequences and genes unique among the three transcriptomes (Fig 1).

**Fig 2:**
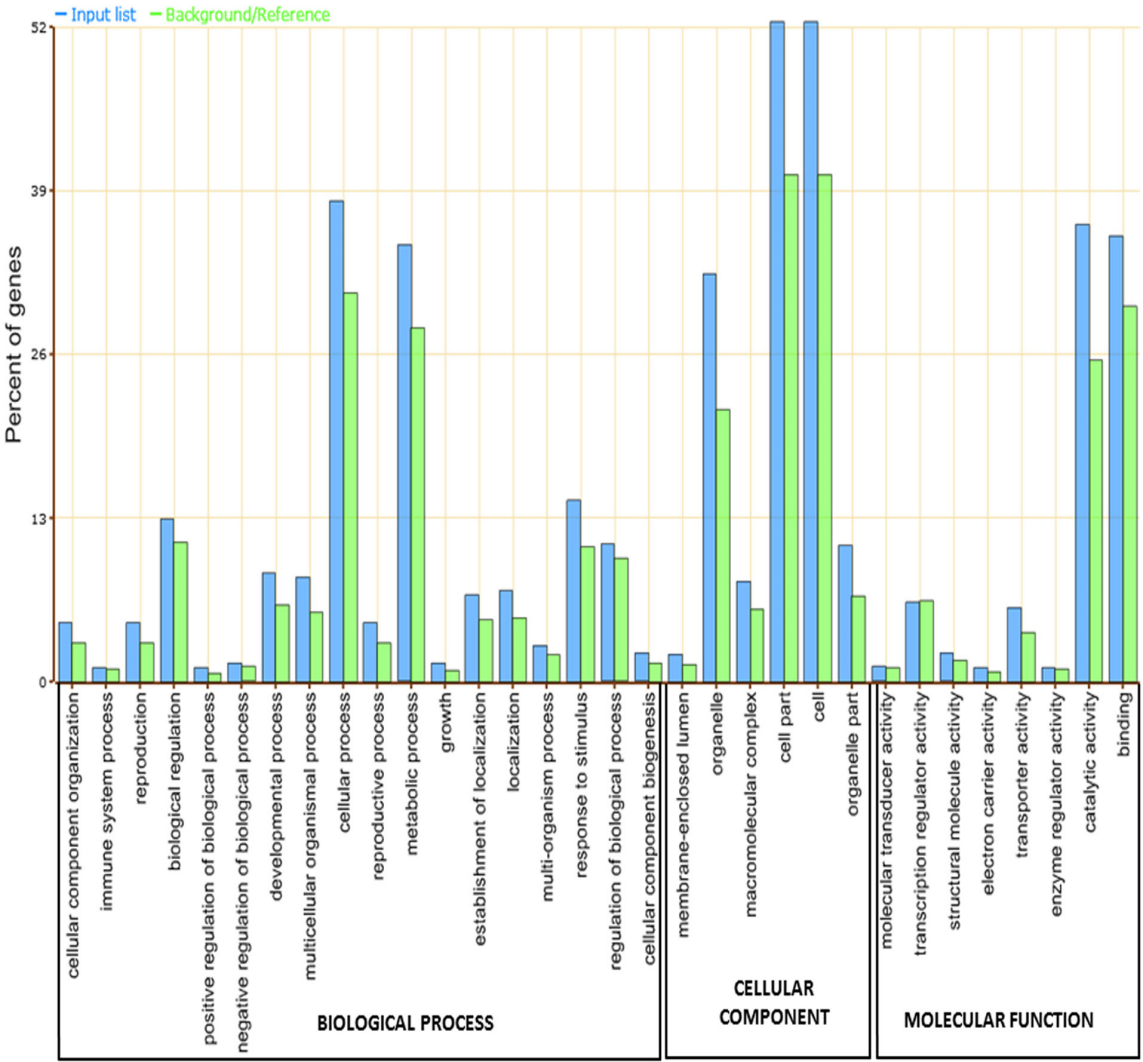
Distribution of the GO categories assigned to the faba bean transcriptome. Unique transcripts were annotated in three categories: biological process, cellular components and molecular functions.

Faba bean transcripts were categorised by predicted function using AgriGO against *A*. *thaliana*. A total of 13,266 transcripts were classified based on the Gene Ontology (GO) hierarchy and assigned at least to one GO term. Transcripts were classified into 401 significant GO terms, of which 237, 83 and 81 belonged to the categories biological process, molecular function and cellular component, respectively. Among the biological process terms, cellular (38.2%) and metabolic processes (34.7%) were the most represented. Other biological processes such as biological regulation (12.9%), developmental process (8.7%), response to stimulus (14.4%) and regulation of biological process (10.9%) were also found (Fig 2). In the molecular function category a significant percentage of genes were assigned to binding and catalytic activity (35.4% and 36.3%, respectively). Other molecular functions found were transcription regulator activity (6.2%) and transporter activity (5.9%) (Fig 2). Finally, the higher number of cellular component annotations fell into cell part (52.4%), organelle (32.4%) and organelle part (10.8%) (Fig 2).

### Sequence similarity of *V*. *faba* transcripts with other plants

The 21,243 faba bean transcripts were analysed for similarity/sequence conservation against the transcript data sets of *A*. *thaliana* and the legume species *M*. *truncatula* and *C*. *arietinum*, using BLASTX. The largest number of faba bean transcripts (17,265 accounting for 81.3%), showed significant similarity with the chickpea unigenes, followed by *Medicago* (15,457 transcripts; 72.8%) and *Arabidopsis* (14,506 transcripts; 68.3%). As reported previously [48] the analysis confirms the close phylogenetic relationship between faba bean, chickpea and *Medicago*. All of them are cool-season legumes, members of the Papilionoid subfamily and diverged from a common ancestor ~60 million years ago.

A tBlastx search was performed with chickpea, whose genome was recently sequenced and annotated, using the chickpea CDS information (GA_v1.0) [49] and the faba bean transcripts described in this study. We found that 17,129 (80.63%) of the faba bean transcripts are conserved in the chickpea transcript data set. Considering the high degree of conservation among legumes, it may be assumed that the assembly of the faba bean transcriptome may further be improved as more sequence data from other species become available.

### Discovery of SNPs and InDels variants

A total of 44,145 putative variants were identified in the transcriptome dataset. After filtering, high confidence differences were obtained resulting in 39,060 SNP and 3,669 Indels (S2 File). The highest number of SNPs detected were C/T (7,320, 18.7%), followed by A/G (6,443, 16.5%), T/C (5,969, 15.3%) and G/A (5,566, 14.2%). The remaining SNP types account for less than 5%. Transitions were the most common SNPs (64.8%) compared to transversions (35.2%).

### Transcripts involved in blight resistance

A comparison between the transcript data derived from the resistant and susceptible genotypes was performed by mapping the sequence reads generated onto the assembled transcripts. Out of the 21,243 transcripts, 850 (4%) showed significant differences between the two genotypes, 393 and 457 transcripts being overexpressed in the resistant and susceptible genotype, respectively. Moreover, 290 transcripts were only unique to the resistant line while 278 were only found in the susceptible line (S3 File).

In order to gain insights into the pathways involved in *Ascochyta* resistance in faba bean, transcripts that consistently showed significant expression differences between resistant and susceptible genotypes were grouped by KEGG IDs. Sixty-five KEGG pathways were identified in the resistant genotype (29H) and 94 in the susceptible one (Vf136). In addition, transcripts involved in 24 pathways related to pathogen resistance in both genotypes were identified, including those involved in biosynthesis of secondary metabolites, ethylene, phenylpropanoid and isoflavonoids.

In the resistant genotype (29H) several transcripts involved in plant-pathogen interaction were differently expressed (S3 File). These genes are leucine rich proteins (LRR) such as RGA2 or FEI1 and plant growth regulators with documented roles in adaptation to abiotic and biotic stresses such as abscisic acid, ethylene, jasmonic acid and salicylic acid. Examples include jasmonate O-methyltransferase, 1-aminocyclopropane-1-carboxylate oxidase, abscisic acid 8'-hydroxylase 3 and salicylic acid carboxyl methyltransferase, as well as several abscisic acid-induced proteins (HVA22 and stress-induced receptor-like kinase) or heat shock proteins (Hsp90, DNAJ/Hsp40 or Hsp70). The signaling pathway genes TGF-beta, p53 and EGF receptor (histone acetyltransferase and rac-like GTP binding protein) as regulators of reactive oxygen species production and cell death in plant species were also represented. Enzymes involved in biosynthesis of secondary metabolites such as chlorogenic acid, scopoletin, suberin and phenylpropanoids; caffeoyl-CoA 3-O-methyltransferase, flavonoids (dihydroflavonol reductase) and chitin elicitor-binding protein were either identified.

A number of genes encoding proteins with essential roles in disease resistance and response were differentially expressed between the resistant and susceptible genotypes. Transcripts encoding NBS-LRR proteins (RGA2 and protein 10), enzymes involved in jasmonate and etilene pathways (methyl jasmonate esterase and 1-aminocyclopropane-1-carboxylate oxidase) and heat shock proteins (Hsp90, Hsp70) were found in different read numbers. Remarkable is the detection of a transcript encoding MLO, a gene conferring broad spectrum powdery mildew resistance in monocots and dicots [50], as well as a MYB-related transcription factor, several pathogenesis-related proteins (PR1a, PR5, a disease resistance response protein 206), a gibberellin induced protein and a CNGC5-like protein. Differential expression of calmodulin and aldehyde dehydrogenase 7a, two key regulators of the plant immune response, was also observed.

### Validation of Transcriptome by RT-qPCR

**Fig 3:**
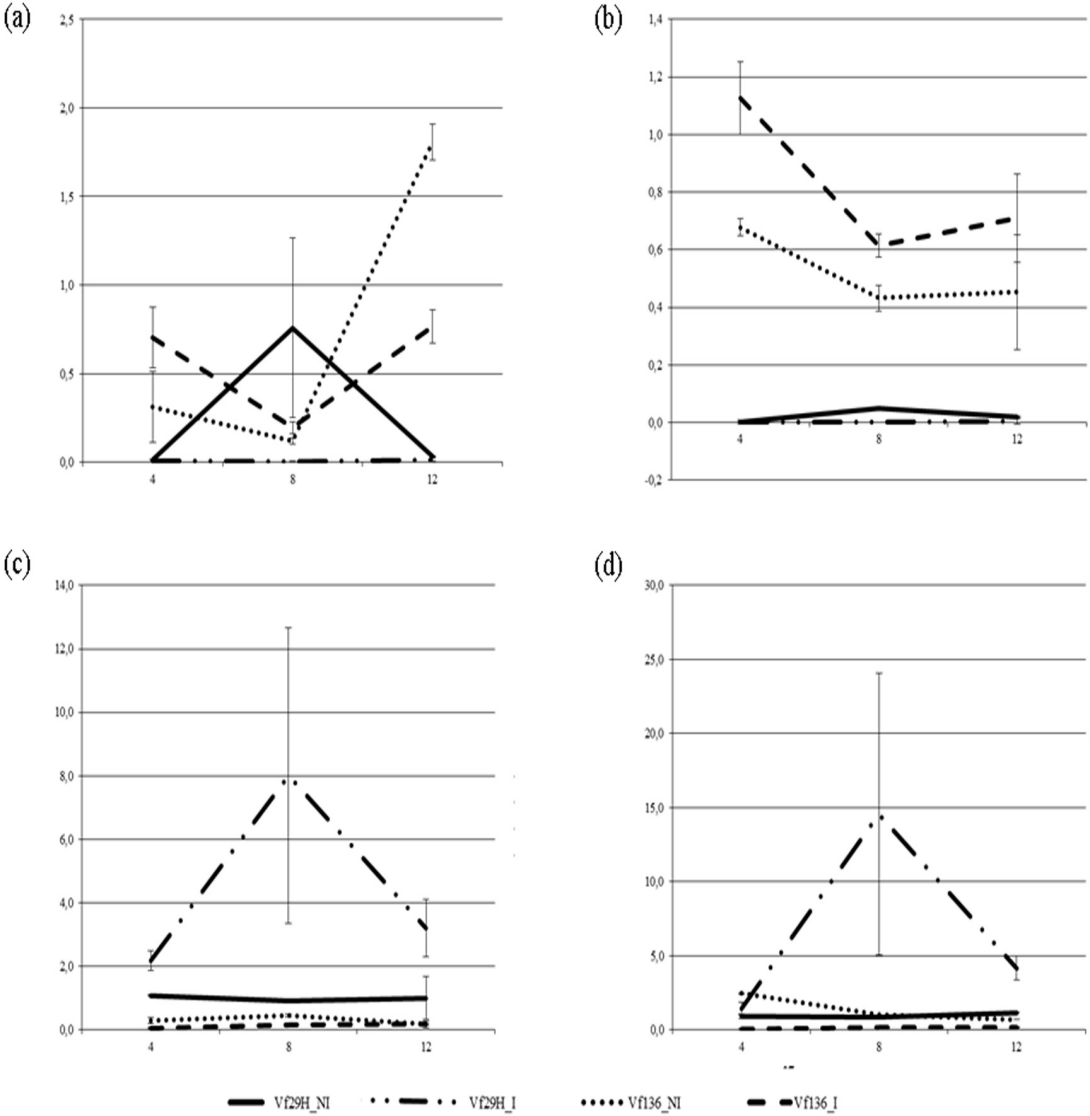
Analysis of the kinetics of 4 transcripts analyzed by RT-qPCR in the resistant and susceptible genotype. (a) LEA-18; (b) Transcription factor NAI1; (c) Jasmonate O-methyltransferase; (d) F-box/LRR-repeat protein At3g59200 analyzed at 4, 8 and 12 hours after inoculation with *A*. *fabae*. Relative mRNA quantification was performed using ACT1 and CYP2, as reference genes for normalization.

To confirm the transcript abundance differences identified by the read counts, expression levels of four transcripts (transcription factor NAI1, LEA-18, Jasmonate O-methyltransferase and F-box/LRR-repeat protein At3g59200, Table 1) were measured by real time RT-qPCR. Both faba bean genotypes were analyzed independently at each time point and condition (inoculated and non-inoculated leaf samples). Transcripts were selected to represent putative candidate genes related to *Ascochyta* resistance and a wide range of expression profiles. NAI1 belongs to the helix-loop-helix (HLH) family and regulates ER body formation. ER bodies are enriched at the epidermis cells to protect plants from pathogens/herbivores that may enter or feed them. This is supported by the fact that leaf wounding triggers local and systemic de novo formation of ER bodies in a jasmonic acid (JA)-dependent manner [51]. LEA proteins have functional properties related to their presumed role as cellular stabilizers under stress conditions [52]. Jasmonic acid is a phytohormone involved in plants response against necrotrophic pathogens [53]. Finally, the F-box genes are involved in the control of many crucial processes such as pathogen resistance, embryogenesis, hormonal responses, etc. Relative transcript levels obtained by RT-qPCR analysis for the selected transcripts were consistent with those previously observed by RNA-seq. The differential expression between the two genotypes was confirmed in all cases (Fig 3) being the expression higher in the inoculated leaves.

## Discussion

Faba bean genetic and genomic studies have been limited by the lack of genomic resources. Transcriptome assemblies allow the detailed comparative analysis across different genera and the discovery of functionally relevant markers. In crops such as faba bean, with a large and poorly characterized genome, comprehensive transcriptome assemblies offer a way to directly access the genes and the causative functional polymorphisms, yielding valuable insights about genome organization [54].

The goal of this study was to generate a comprehensive faba bean transcriptome assembly with the final aim of detecting potential resistance genes for *Ascochyta fabae*. For this purpose, sequences from three different data sets were combined: newly obtained transcripts from Illumina sequencing, those obtained from FLX/454 reads [23] and the ESTs sequences available at public databases. The completeness and quality of the new assembly was superior to earlier faba transcriptome assemblies [23, 36]. For instance, when the datasets were analyzed individually, a wide range of counts were reported: 85,844 transcripts from 304,680 FLX/454 reads [23] and 98,195 transcripts from 30,907,202 Illumina reads. Hybrid assembly using a combination of different datasets was previously shown to be superior to that generated from a single sequencing platform [55]. Drawbacks of a single sequencing platform can be compensated by different characteristics of sequences obtained from other platforms, and the combination of both may help to correct sequence errors/biases improving the quality of draft assembly [55]. The enriched transcriptome obtained in the present study increased the number of annotated genes by 27.8% as compared with the previous report [23]. Although a single pooled sample was used for each genotype and condition, the differences in gene expression of four tested transcripts determined by RT-qPCR were consistent with those obtained by RNA-seq, confirming the validity of the expression data. Further research is required to determine the potential function of the identified candidate genes in faba bean disease resistance.

BLAST searches against public databases allowed annotation for the 98,195 faba bean transcripts, yielding a set of 21,243 (54.21%) non-redundant transcripts with an orthologue gene ID. As reported in other legumes crops [56], comparison across species favors understanding of the biology and identification of genes in under-studied crops such as faba bean. This information may facilitate gene expression analysis and deliver evidence about gene content and function both of particular interest for the identification of candidate genes and the development of molecular markers.

The number of unigenes described in this study (21,243) is close to those estimated for diploid plants such as *A*. *thaliana* (25,000) [57] or *C*. *arietinum* (28,269) [49]. Interestingly, 270 transcripts likely encoding complete proteins did not show significant homology with any other sequence in the database, indicating that these transcripts may represent faba bean specific genes. Lineage and species-specific genes have been identified previously in other plant species, including legumes [58, 59]. These genes could be interesting for further functional studies and may reveal novel legume-specific pathways. Moreover, knowledge on these genes may help to dissect species-specific cellular processes and to understand evolutionary processes such as speciation and adaptation.

The functional annotation obtained in faba bean was similar to that of *C*. *arietinum* [60] in terms of GO descriptions. The 14.4% of the annotated genes felt into “response to stimulus” subcategory. This result could contribute to our understanding of the global transcriptional changes occurring during infection by *A*. *fabae*. However, changes at the gene expression level are not necessarily a direct indication of the involvement of a gene in a biological process. Therefore, further genetic and functional analyses of differentially expressed transcripts are required to understand the biological significance of these changes in gene expression and their role in plant immune response.

Analysis of sequence conservation might help in the transfer knowledge from model plants to faba bean for functional genomic studies. As expected, lower similarity of faba bean transcripts was found with *Arabidopsis* compared with model legume species *Medicago* and chickpea. As previously reported [16, 18, 49], our results confirm the close phylogenetic relationship and genome conservation among the three galegoid cool-season legumes that derived from a common ancestor [48]. A large number (81%) of predicted faba bean proteins showed significant similarity with chickpea, indicating that their function might be conserved. Given the high degree of conservation among legumes, assembly of the faba bean transcriptome could be further improved as additional legume sequence data become available.

The present study identified a large set of potential SNPs between the lines 29H and Vf136 (44,146). Almost double rates of transitions in comparison with transversions were identified, comparable with results obtained in other plant species [61, 62]. Conversion of these SNPs into GoldenGate [63] or KASP genotyping assays (http://www.lgcgroup.com/products/kasp-genotyping-chemistry/) will provide a low-cost and high-throughput marker genotyping system for accelerating their use in genetics and breeding programmes. Mapping and colocalization of these SNPs in previously reported *A*. *fabae* resistance QTLs [13, 15, 17] will validate the functional relationship of these candidate genes and their future application in molecular breeding approaches.

Since the two sequenced genotypes showed differential resistance to *A*. *faba*e, and as DNA libraries were obtained from inoculated and non-inoculated leaves, genes with differential expression were identified in both genotypes. The results provided by this study are of interest to understand the transcriptional regulation of defense-associated genes as a first step toward understanding faba bean resistance to Ascochyta blight and improvement of disease resistance in plants [64]. Nevertheless, the nature of the signaling systems involved in resistance to major diseases in faba bean is still not well understood. The necrotrophic nature of *A*. *fabae* further complicates the elucidation of the resistance mechanism acting against this pathogen. As a result, only few studies have been reported to reveal the genes and metabolic pathways involved in this resistance [31, 65, 66]. In other pathosystems, however, a wide range of defense responses are known to be induced. These include preformed structural and chemical components, activation of the phytoalexin biosynthetic pathway, production of PR proteins, cell wall reinforcement mediated by hydrogen peroxide and detoxification of fungal toxins [66]. Our results suggest that several of these mechanisms may contribute to resistance to *A*. *fabae* in faba bean line 29H.

After perception and recognition of a pathogen, constitutive basal defense mechanisms lead to an activation of complex signaling cascades of defense. Thus, ion channels and kinase cascades are activated, reactive oxygen species (ROS), phytohormones like abscisic acid (ABA), salicylic acid (SA), jasmonic acid (JA), and ethylene (ET) accumulate in concert to reduce infection, and a reprogramming of the genetic machinery lead to defense reactions to minimize the biological damage caused by the stress [53, 67, 68]. The comparison between the resistant (29H) and the susceptible (Vf136) genotypes revealed significant differences in the expression of several genes related to these defence mechanisms. Thus, several phytoalexins (Dihydroflavonol-4-reductase) and a chitin elicitor-binding protein (CEBiP) were only expressed in line 29H (S3 File). Chitin recognition results in the activation of defense signaling pathways and CEBiP is known to plays a critical role in plant cells to mediate chitin perception and plant disease resistance [69]. There was also evidence of alterations in cell wall metabolism indicated by overexpression of cellulose synthase biosynthesis genes. Other genes involved in the synthesis of methyl-jasmonate (jasmonate-O-methiltransferase), ethylene (1-O-aminociclopropano-1-carboxilato), abcisic acid (abcisic acid 8-hidroxylase3) and salicylic acid (adenosyl-L-methionine: benzoic/salicylic acid carboxyl methyltransferase) were overexpressed or expressed only in the resistant genotype. Additional examples are provided by the ACC oxidase (only expresses in the resistance line) that catalyzes the final step of ethylene synthesis after induction by biotic or abiotic stresses (S3 File). Some of these findings were verified by RT-qPCR. Thus the enzyme Jasmonate O-methyltransferase, and a F-box/LRR-repeat protein were clearly activated only in line 29H. Conversely, two differentially expressed genes, NAI1 and LEA-18, which were found to be expressed in the susceptible faba bean line were not activate in the resistant line. Differentially expressed genes involved in these pathways point towards potential *Ascochyta* disease-resistance genes for future use in precision breeding.

Several pathogenesis related (PR) proteins were as well mainly expressed in the resistant genotype, suggesting that they may be key players in the faba bean-ascochyta interaction. PR proteins are indicators of a gene-for-gene resistance, associated with an immune response known as systemic acquired resistance (SAR) [70, 71]. Resistance (*R*) genes are important for plant breeding purposes being ultimately responsible for activation of plant defense mechanisms [64]. Among these, the nucleotide binding site-leucine rich repeat (NBS-LRR) class is the most abundant and found in all types of plants [72]. Our analysis identified a number of CC-NBS and NBS-LRR resistance proteins (such as RGA2, FEI1) that were highly expressed in the resistant parent. In addition, genes involved in secondary metabolism were also found to be expressed upon fungal infection. These metabolites are responsible in higher plants for generating metabolic intermediates directed into several pathways, including the lignin and flavonoid pathways [73, 74]. Legumes utilize flavonoids, notably isoflavones and isoflavanones, to defend themselves against physical injury and pathogens. Many of these effects appear to be related to their ability to modulate cell-signaling pathways [75].

## Conclusions

The present study contributes a non-redundant set of 21,243 transcripts in *V*. *faba* and provides a global view of genes expressed during the interaction with *A*. *fabae*. The assembly described here is the most comprehensive transcript database developed so far in this crop. Data set analysis revealed important features of the faba been transcriptome such as gene annotation, assignment of functional categories and identification of SNPs. The large number of SNPs and InDels identified offer a cost-effective way to further develop functional markers for assisting breeding purposes. Furthermore, transcripts identified under specific categories like response to stimulus and enzyme classification, represent a valuable resource for faba bean crop improvement by identifying stress-response genes and genes involved in key metabolic pathways.

Different mechanisms and pathways involved in the resistance to pathogens such as chitin recognition, production of phytoalexins and PR proteins were present. We also identified putative candidate genes related to pathogen resistance that will be future targets for molecular breeding of *A*. *fabae* resistance in faba bean. The next steps will include the identification of polymorphisms in the candidate resistance genes to facilitate gene mapping and validation in forthcoming marker-assisted breeding programs.

## Supporting Information

S1 File Transcripts collection annotated using Full-LengtherNext based on orthologue ID. (XLS)

S2 File Transcriptome nucleotide variations. List of nucleotide variation (SNPs and InDels) obtained by mapping separately the reads from each genotype against the tentative transcripts.
(XLSX)

S3 File List of transcripts differentially expressed between resistant and susceptible genotypes. The reads derived from both genotypes were mapped onto the assembled transcripts.
(XLSX)
